# Glances at the Hospitals

**Published:** 1901-11-16

**Authors:** 


### GLANCES AT THE HOSPITALS.

### KENT AND CANTERBURY HOSPITAL.

The ordinary receipts amounted to £3,379 and the ordinary expenditure to £4,224, showing an adverse balance of £845. For the previous year there was a balance on the wrong side of £643 and to meet this India Stock had to be sold. While commending the Governors for meeting their responsibilities in this way, it is not a course which can be pursued for many consecutive years without seriously crippling their resources, and if subscriptions be not forthcoming the expenditure must be reduced. Of this the Governors seem well aware, and a committee has been appointed to overhaul every item of expenditure. The workpeople's contributions are put down at £39 17s., and in view of the sums raised in some infirmaries it ought to be possible to increase this amount. In other infirmaries there is a wage limit, and no one receiving above a certain sum is eligible for free treatment. There were 69 patients in the hospital at the beginning of the year and 783 were admitted, making a total of 853. Of these 719 were discharged cured or relieved, 10 not relieved, 4 for various reasons, and 59 died. The average residence in the house was 32½ days, which is considered a long time nowadays. The out-patients numbered 1,003, and the average number of days each out-patient remained on the books was 51. The medical and surgical diseases are divided into groups, and in all cases the numbers cured, relieved, not relieved, and died are given. There were 24 cases of acute rheumatism of which 23 recovered and 1 was relieved. Of 20 cases of enteric fever 19 recovered and 1 died. The statistics are so good that it is a pity a table showing the anæsthetics used is not printed.

### QUEEN'S HOSPITAL, BIRMINGHAM.

The income was £9,583 and the expenditure was £10,970, but part of the latter was extraordinary expenditure on the building. As the interest on their invested capital only amounts to about £1,000 it is necessary to raise £10,000 a year. On last year's working there is a deficit of £1,386. The average residence of each in-patient was 20·5 days, and the cost was £3 2s. 7d. The daily average number resident was 119, and the death rate was 1·6 per bed. When the Birmingham University was established by Royal Charter the medical school was absorbed into the University, and the Queen's Hospital is permanently attached to the latter as a clinical institution. The total number of in-patients was 2,236, and of the out-patients 26,725, making a grand total of 28,961, and affording an extensive and varied field for the purposes of clinical teaching. There were 67 cases of enteric fever, of these 40 were cured, 8 died, and 19 remained under treatment on the date of the report. Of 55 cases of pneumonia, 46 were cured, 2 relieved, and 7 died. In Mr. May's ward nephrectomy was performed twice for pyonephrosis, and both cases were cured. Trephinings was also twice performed for mastoid abscess, and both cases recovered. In Mr. Lloyd's wards appendectomy was performed 18 times, with 3 deaths, 2 of the fatal cases being suppurative, and 1 relapsing. Herniotomy and radical cure were performed 34 times, with 3 deaths. In the eye department 235 operations were performed. As an instance of the growth of hospitals and the ever-increasing amount of work done in them, it may be stated that in 1870 there were only 1,450 in-patients, and 15,445 out-patients; whereas last year the in-patients had risen to 2,236, and the out-patients to 26,725. A table showing the nature of the anæsthetics used would be a desirable addition to next year's report.

GLASGOW ROYAL INFIRMARY.

The ordinary revenue amounted to £27,352, and the ordinary expenditure to £35,910, showing a deficiency on this account of £8,558 ; but the extraordinary revenue amounted to £19,626, and the extraordinary expenditure to £3,564. Treating the two accounts as one, there was a balance of £7,502 in favour of the infirmary. Some parts of this infirmary are over 100 years of age, and it has been wisely decided to reconstruct the whole building. At the beginning of the year there were 534 patients in residence, and 6,268 were admitted during the year, making a total of 6,802 under treatment. Of these, 6,256 were treated to a conclusion, and there were 691 deaths. The average duration of treatment in the infirmary was 31 days. The medical and surgical tables are carefully compiled, and show the numbers treated for the various diseases and the results. There were 90 cases of consumption, and of these 6 were cured, 25 relieved, 31 discharged for other reasons, and 28 died. It is to be hoped that the new infirmary will contain special arrangements for the open-air treatment of this disease. Acute rheumatism was represented by 55 cases; 40 of these were cured, 13 relieved, 1 was discharged not relieved, and 1 died. Of 166 cases of pneumonia 114 were cured, 3 relieved, and 44 died. Of 40 cases of appendicitis 33 were cured, 1 was relieved, and 3 died. Of 118 amputations, 97 of the patients recovered and 21 died. The infirmary contains a resident medical staff of 14, and there are 145 nurses. A medical school is attached to it.

MANCHESTER ROYAL INFIRMARY.

The income of the infirmary and the convalescent hospital amounted to £25,729, and the expenditure to £25,097. During the year the daily average of occupied beds was 264, and the cost per bed occupied was £56 6s. There were 4,272 in-patients admitted, and 253 remained from the previous year, making a total of 4,525. Of these 477 were cured, 465 relieved, 1,464 were transferred to the convalescent homes, 12 to Buxton, 9 to the fever hospital; 1,166 were made out-patients, 128 were discharged at own request, 77 for other reasons, and 379 died, but of the latter 77 died within 24 hours of admission. The out-patients reached the high figure of 38,985. The returns of medical and surgical in-patients are practically useless, as only bald figures are given, and there is neither an operation table nor a post-mortem examination table, nor a table showing the nature of the anæsthetics used.

